# Supplementary material for: Tgfβ signaling is critical for maintenance of the tendon cell fate
Source: eLife. 2020 Jan 21;9:e52695. doi: 10.7554/eLife.52695 (PMC7025861; doi:10.7554/eLife.52695)
Supplement: Supplementary file 1. — See also Figure 6A for the tSNE plots of the sample. (A) Top 25 genes highly expressed in the tenocyte cluster relative to other clusters in the P7 wild-type tendon sample (≥1.5 fold change, adjusted p<0.05). (B) Top 25 genes highly expressed in the dedifferentiated mutant cell cluster relative to other clusters in the P7 Tgfbr2;ScxCre mutant tendon sample (≥1.5 fold change, adjusted p<0.05). [file elife-52695-supp1.docx]

**Supplementary File 1. Signature genes in tenocytes and dedifferentiated mutant cells in comparison with other clusters.** See also Figure 6A for the tSNE plots of the sample.

(A) Top 25 genes highly expressed in the tenocyte cluster relative to other clusters in the P7 wild-type tendon sample (≥1.5-fold change, adjusted *p*<0.05).

| Gene symbol | Gene name | Fold change |
| --- | --- | --- |
| *Angptl7* | Angiopoietin like 7 | 30.3 |
| *Car9* | Carbonic anhydrase 9 | 18.5 |
| *Col11a2*^#^ | Collagen, type XI, alpha 2 | 16.4 |
| *Scx*^#^ | Scleraxis | 14.9 |
| *Wif1* | Wnt inhibitory factor 1 | 13.5 |
| *Sema3b* | Sema domain, immunoglobulin domain (Ig), short basic domain, secreted, (semaphorin) 3B | 12.7 |
| *Col2a1* ^δ^ | Collagen, type II, alpha 1 | 12.6 |
| *Fmod^#^* | Fibromodulin | 11.9 |
| *Matn4* | Matrilin 4 | 10.1 |
| *Cgref1* | Cell growth regulator with EF hand domain 1 | 9.9 |
| *Col11a1*^#^ | Collagen type XI alpha 1 | 9.3 |
| *1500015O10Rik* | RIKEN cDNA 1500015O10 gene | 9.2 |
| *Cpxm2* | Carboxypeptidase X 2 (M14 family) | 9.1 |
| *Clip2* | Cartilage intermediate layer protein 2 | 8.3 |
| *Pdgfrl*^δ^ | Platelet-derived growth factor receptor-like | 7.3 |
| *Itgbl1* | Integrin, beta-like 1 | 7.0 |
| *Col1a1*^#^ | Collagen, type I, alpha 1 | 6.9 |
| *Col1a2*^#^ | Collagen, type I, alpha 2 | 6.9 |
| *Cpz* | Carboxypeptidase Z | 6.7 |
| *Kera* ^δ^ | Keratocan | 6.6 |
| *P4ha1*^δ^ | Procollagen-proline, 2-oxoglutarate 4-dioxygenase (proline 4-hydroxylase), alpha 1 polypeptide | 6.2 |
| *Abi3bp* | ABI gene family, member 3 (NESH) binding protein | 5.9 |
| *Crabp1* | Cellular retinoic acid binding protein I | 5.8 |
| *Tnmd*^#^ | Tenomodulin | 5.7 |
| *C1qtnf3* | C1q and tumor necrosis factor related protein 3 | 5.4 |

Note:

1. #=Tendon differentiation or specific marker; δ=genes related to tendons.
2. Note that the expression level detected for *Scx* also included that of *ScxGFP*, and therefore do not reflect the expression level of endogenous *Scx*.

(B) Top 25 genes highly expressed in the dedifferentiated mutant cell cluster relative to other clusters in the P7 *Tgfbr2;ScxCre* mutant tendon sample (≥1.5-fold change, adjusted *p*<0.05).

| Gene symbol | Gene name | Fold change |
| --- | --- | --- |
| *Apod* | Apolipoprotein D | 12.8 |
| *Cxcl14* | Chemokine (C-X-C motif) ligand 14 | 10.3 |
| *Ptn* | Pleiotrophin | 9.2 |
| *Itm2a* | Integral membrane protein 2A | 6.8 |
| *Cxcl12* | Chemokine (C-X-C motif) ligand 12 | 6.5 |
| *Serping1* | Serine (or cysteine) peptidase inhibitor, clade G, member 1 | 6.1 |
| *Clec3b* | C-type lectin domain family 3, member b | 6.1 |
| *Sfrp2* | Secreted frizzled-related protein 2 | 6.0 |
| *Plpp3* | Phospholipid phosphatase 3 | 5.5 |
| *Igfbp3* | Insulin-like growth factor binding protein 3 | 5.5 |
| *Kera* | Keratocan | 5.5 |
| *Dlk1* | Delta-like 1 homolog (Drosophila) | 5.0 |
| *Gsn* | Gelsolin | 4.9 |
| *Ly6a*^ϕ^ | Lymphocyte antigen 6 complex, locus A | 4.7 |
| *Igf1* | Insulin-like growth factor 1 | 4.7 |
| *Dpt* | Dermatopontin | 4.5 |
| *Cd34*^ϕ^ | CD34 antigen | 4.3 |
| *H19* | Cadherin 19, type 2 | 4.2 |
| *Ly6c1* | Lymphocyte antigen 6 complex, locus C1 | 4.1 |
| *Ifitm3* | Interferon induced transmembrane protein 3 | 4.0 |
| *Mgst1* | Microsomal glutathione S-transferase 1 | 3.9 |
| *Lum* | Lumican | 3.7 |
| *Mgp* | Matrix Gla protein | 3.4 |
| *Spon2* | Spondin 2, extracellular matrix protein | 3.2 |
| *Igf2* | Insulin-like growth factor 2 | 3.1 |

Note:

1. ϕ=genes in which the encoding protein expression in the mutant cells was confirmed by immunostaining.
2. The dedifferentiated mutant cell cluster was also identified based on the absence of tendon markers on the significant gene list.
