# Supplementary material for: Tgfβ signaling is critical for maintenance of the tendon cell fate
Source: eLife. 2020 Jan 21;9:e52695. doi: 10.7554/eLife.52695 (PMC7025861; doi:10.7554/eLife.52695)
Supplement: Supplementary file 3. — A complete list of differentially expressed genes (≥2 fold change, p<0.05) used for the analysis is available in Supplementary file 2. [file elife-52695-supp3.docx]

**Supplementary File 3. Gene Ontology (GO) term enrichment of differentially expressed genes in P7 *Tgfbr2;ScxCre* mutant cells compared with P7 wild-type tenocytes.** A complete list of differentially expressed genes (≥2-fold change, adjusted *p*<0.05) used for the analysis is available in Supplementary File 2.

1. **Upregulated genes**

| GO ID | GO terms (biological process) | *p*-value | Genes annotated to the term | Count |
| --- | --- | --- | --- | --- |
| GO:2000146 | negative regulation of cell motility | 4.77E-07 | *Cxcl12/Col3a1/Ptn/Nbl1/Apod/Sfrp2/Igfbp3/Apoe/Igfbp5* | 9 |
| GO:0061041 | regulation of wound healing | 1.34E-06 | *Serpine2/Cd34/Serping1/Gsn/Plpp3/Apoe/Anxa1* | 7 |
| GO:0042060 | wound healing | 1.38E-06 | *Serpine2/Cd34/Serping1/Col3a1/Gsn/Col5a1/Igf1/Apoe/*  *Anxa1/Naca* | 10 |
| GO:0009611 | response to wounding | 1.61E-06 | *Serpine2/Cd34/Serping1/Col3a1/Gsn/Col5a1/Igf1/Apoe/*  *Mmp2/Anxa1/Naca* | 11 |
| GO:0043567 | regulation of insulin-like growth factor receptor (IGFR) signaling pathway | 2.18E-06 | *Igf1/Igfbp3/Igfbp5/Igfbp4* | 4 |
| GO:0031099 | regeneration | 9.23E-06 | *Cxcl12/Igf1/Apoe/Mmp2/Anxa1/Naca* | 6 |
| GO:0050900 | leukocyte migration | 2.83E-05 | *Cd34/Cxcl12/Nbl1/Apod/Anxa1/Cxcl14/Mmp14/Rps19* | 8 |
| GO:0048771 | tissue remodeling | 7.54E-05 | *Dlk1/Igf1/Igfbp5/Mmp2/Anxa1/Mmp14* | 6 |

**Supplementary File 3 (Continued). Gene Ontology (GO) term enrichment of differentially expressed genes in P7 *Tgfbr2;ScxCre* mutant cells compared with P7 wild-type tenocytes.**

1. **Downregulated genes**

| GO ID | GO terms (biological process) | *p*-value | Genes annotated to the term | Count |
| --- | --- | --- | --- | --- |
| GO:0030199 | collagen fibril organization | 1.86E-17 | *Col11a2/Scx/Col2a1/P4ha1/Loxl2/Col11a1/Col1a1/*  *Col1a2/Lox/P3h4/Serpinh1* | 11 |
| GO:0030198 | extracellular matrix organization | 5.57E-16 | *Col11a2/Scx/Col2a1/P4ha1/Loxl2/Col11a1/Mfap4/Col1a1/*  *Col1a2/Lox/Abi3bp/Creb3l1/Comp/P3h4/Tgfbi/Serpinh1* | 16 |
| GO:0070208 | protein heterotrimerization | 1.44E-08 | *Col1a1/Col1a2/Col6a2/Col6a1/C1qtnf6* | 5 |
| GO:0032964 | collagen biosynthetic process | 4.92E-08 | *Scx/Col1a1/Creb3l1/P3h4/Serpinh1/P3h3* | 6 |
| GO:0032963 | collagen metabolic process | 8.63E-08 | *Scx/Mfap4/Col1a1/Creb3l1/P3h4/Serpinh1/P3h3* | 7 |
| GO:0070206 | protein trimerization | 1.60E-07 | *Col1a1/Col1a2/C1qtnf3/Col6a2/Col6a1/C1qtnf6* | 6 |
| GO:0018126 | protein hydroxylation | 1.79E-06 | *P4ha1/Crtap/P3h4/P3h3* | 4 |
| GO:0001501 | skeletal system development | 6.72E-06 | *Col11a2/Scx/Col2a1/Loxl2/Col11a1/Col1a1/Col1a2/Sparc/*  *Comp/Vkorc1/Serpinh1* | 11 |
