# Supplementary material for: Tgfβ signaling is critical for maintenance of the tendon cell fate
Source: eLife. 2020 Jan 21;9:e52695. doi: 10.7554/eLife.52695 (PMC7025861; doi:10.7554/eLife.52695)
Supplement: Transparent reporting form [file elife-52695-transrepform.docx]

***eLife’s* transparent reporting form**

We encourage authors to provide detailed information *within their submission* to facilitate the interpretation and replication of experiments. Authors can upload supporting documentation to indicate the use of appropriate reporting guidelines for health-related research (see [EQUATOR Network](http://www.equator-network.org/%20)), life science research (see the [BioSharing Information Resource](https://biosharing.org/)), or the [ARRIVE guidelines](http://www.plosbiology.org/article/info:doi/10.1371/journal.pbio.1000412) for reporting work involving animal research. Where applicable, authors should refer to any relevant reporting standards documents in this form.

If you have any questions, please consult our Journal Policies and/or contact us: [editorial@elifesciences.org](mailto:editorial@elifesciences.org).

**Sample-size estimation**

- You should state whether an appropriate sample size was computed when the study was being designed
- You should state the statistical method of sample size computation and any required assumptions
- If no explicit power analysis was used, you should describe how you decided what sample (replicate) size (number) to use

Please outline where this information can be found within the submission (e.g., sections or figure legends), or explain why this information doesn’t apply to your submission:

Most findings obtained in this study are qualitative. The sample size collected is thus based our experiences on similar studies performed in the laboratory and in the literature. The mutation in this study is 100% penetrant, and we usually collected at least 3 experimental pups per time point to ensure reproducibility of results. A similar number of control pups were included	in each	of those time points.

For the qualitative studies

(a) In situ hybridization and histological staining: cryosections from 2-4 pups were reviewed to confirm the marker expression.

(b) TEM analysis: 3 pups per time point were processed for analysis.

(c) scRNA-Seq: tendons were collected and pooled from 2 pups.

For the quantitative studies

(a) Colony-forming unit assay: each data point represents the mean of duplicate plates from the 3-5 separate experiments.

(b) Viral reintroduction of *Tgfbr2*: 3 pups for each data point.

The number of animals used for each experiment is specified in the Materials & Methods section as well as figure legends wherever appropriate.

**Replicates**

- You should report how often each experiment was performed
- You should include a definition of biological versus technical replication
- The data obtained should be provided and sufficient information should be provided to indicate the number of independent biological and/or technical replicates
- If you encountered any outliers, you should describe how these were handled
- Criteria for exclusion/inclusion of data should be clearly stated
- High-throughput sequence data should be uploaded before submission, with a private link for reviewers provided (these are available from both GEO and ArrayExpress)

Please outline where this information can be found within the submission (e.g., sections or figure legends), or explain why this information doesn’t apply to your submission:

Number of samples used to generate data is specified in the Materials & Methods section and in the figure legends (Figure 5, Figure 7). Of note, compared with the initial submission, we now include sample number and replication details in some parts of Materials & Methods section (wherever appropriate).

**Statistical reporting**

- Statistical analysis methods should be described and justified
- Raw data should be presented in figures whenever informative to do so (typically when N per group is less than 10)
- For each experiment, you should identify the statistical tests used, exact values of N, definitions of center, methods of multiple test correction, and dispersion and precision measures (e.g., mean, median, SD, SEM, confidence intervals; and, for the major substantive results, a measure of effect size (e.g., Pearson's r, Cohen's d)
- Report exact p-values wherever possible alongside the summary statistics and 95% confidence intervals. These should be reported for all key questions and not only when the p-value is less than 0.05.

Please outline where this information can be found within the submission (e.g., sections or figure legends), or explain why this information doesn’t apply to your submission:

Statistical analysis methods are described in the Materials & Methods section. Mean values, SD and *p*-value are also specified in Figure 5, Figure 6, Figure 7, Tables 1-3 and Supplementary Files 1-3.

(For large datasets, or papers with a very large number of statistical tests, you may upload a single table file with tests, Ns, etc., with reference to sections in the manuscript.)

**Group allocation**

- Indicate how samples were allocated into experimental groups (in the case of clinical studies, please specify allocation to treatment method); if randomization was used, please also state if restricted randomization was applied
- Indicate if masking was used during group allocation, data collection and/or data analysis

Please outline where this information can be found within the submission (e.g., sections or figure legends), or explain why this information doesn’t apply to your submission:

Groups were allocated according to experimental conditions and designated time-points. The specific grouping for each study is indicated in the Materials & Methods, Result sections and the figure legends.

**Additional data files (“source data”)**

- We encourage you to upload relevant additional data files, such as numerical data that are represented as a graph in a figure, or as a summary table
- Where provided, these should be in the most useful format, and they can be uploaded as “Source data” files linked to a main figure or table
- Include model definition files including the full list of parameters used
- Include code used for data analysis (e.g., R, MatLab)
- Avoid stating that data files are “available upon request”

Please indicate the figures or tables for which source data files have been provided:

All data generated or analyzed during this study are included in the manuscript and Supplementary Files.

A complete list of differentially expressed genes used for the analysis of Table 1, Table 2 and Supplementary File 3 is attached as additional file named “Supplementary File 2-DEGs in P7 mutant cells. xlsx”.

Single cell RNA-Seq data has been deposited onto GEO under accession code GSE139558.

Reviewers link to GEO data:

https://www.ncbi.nlm.nih.gov/geo/query/acc.cgi?acc=GSE139558.

Token to access the data for reviewers: elkhqeqmppgpbgl
